# Leukemic retinopathy presenting as concurrent bilateral subhyaloid hemorrhage and subarachnoid hemorrhage in a patient with acute monocytic leukemia: a case report

**DOI:** 10.1186/s13256-022-03700-4

**Published:** 2022-12-17

**Authors:** MohammadJavad Ghanbarnia, Sadegh Sedaghat, Seyed Ahmad Rasoulinejad

**Affiliations:** 1 Student Research Committee, Babol University of Medical Sciences, Babol, Iran; 2 Cancer Research Center, Health Research Institute, Babol University of Medical Sciences, Babol, Iran; 3 Department of Internal Medicine, Rouhani Hospital, Babol University of Medical Sciences, Babol, Iran; 4 Department of Ophthalmology, Rouhani Hospital, Babol University of Medical Sciences, Babol, Iran

**Keywords:** Subhyaloid hemorrhage, Retinal hemorrhage, Leukemic retinopathy, Subarachnoid hemorrhage, Acute myelogenous leukemia, AML, Thrombocytopenia, Case report

## Abstract

**Background:**

Ophthalmic manifestations are common in patients with leukemia, developing in nearly 50% of cases. Intracranial hemorrhage is another potentially fatal complication of leukemia. In this case report, we aim to present a challenging case that involves both ophthalmic and intracranial manifestations in an individual with acute monocytic leukemia.

**Case presentation:**

A 36-year-old Persian male presented to the emergency room with complaints of fever, headache, and bilateral blurred vision. The patient had been diagnosed with acute monocytic leukemia 3 months prior and had undergone four sessions of induction chemotherapy, the last of which was 10 days prior to admission. The patient was admitted to the internal medicine service, and initial lab studies confirmed pancytopenia, including severe neutropenia, anemia, and thrombocytopenia. Subarachnoid hemorrhage in the left frontal lobe was detected through spiral brain computed tomography scan. Ophthalmic examination revealed visual acuity of light perception in the right eye and 3-m finger count in the left eye. Fundus examination revealed bilateral peripapillary subhyaloid and intraretinal hemorrhages, confirming leukemic retinopathy. The patient showed significant improvement in visual acuity and hemorrhage resolution through conservative treatment and regular follow-ups after 3 months.

**Conclusion:**

Simultaneous subarachnoid hemorrhage and bilateral subhyaloid hemorrhages seemed to have occurred as a result of pancytopenia. Management approach of ophthalmic manifestations of leukemia involves interdisciplinary cooperation and should be individualized on the basis of the patients’ underlying medical condition.

## Background

Acute myelogenous leukemia (AML) accounts for the majority of leukemia cases in adults, composing almost 80% of all diagnosed cases [1, 2]. Leukemia is a highly heterogeneous disease that can directly or indirectly effect various tissues and organs in the body, among which the eyes and the brain are two of the most critical. Leukemic manifestations in these two organs are usually accompanied by sudden onset and rapid progression. As a result, in many instances, ophthalmic and cerebrovascular manifestations of leukemia lead to adverse outcomes and poor quality of life. Ophthalmic manifestations of leukemia are relatively common, occurring in nearly half of all patients diagnosed with leukemia [3, 4]. Ophthalmic manifestations are significantly more frequent in patients with AML, presenting in 68% of patients [5]. These present as either primary involvement by direct infiltration of leukemic cells, or secondary involvement as a result of hematologic disturbances, including thrombocytopenia and anemia [2–5]. However, secondary involvements constitute the major proportion of ophthalmic manifestations [2–5]. Retina, among all ocular tissues, is the tissue most commonly affected by hematologic disturbances, and leukemic retinopathy has been estimated to effect up to 70% of patients at some point during the course of leukemia [2–6]. Leukemic retinopathy presenting as retinal hemorrhage occurs at any layer in the posterior pole, and in 20% of cases, subhyaloid hemorrhage can cause vision-threatening complications [5, 6]. Even though ophthalmic manifestations have been well established in the literature, their co-occurrence with cerebrovascular manifestations have not been well presented.

Intracranial hemorrhage (ICH) is another critical manifestation of acute leukemia that most commonly occurs as a result of underlying hematologic disturbance such as thrombocytopenia. Owing to its acute onset and rapid progression, ICH in patients with leukemia can be potentially fatal. ICH is far less frequent than ophthalmic manifestations, occurring in around 6.3% of patients with AML [7]. Subarachnoid hemorrhage (SAH) constitutes around 21% of all ICH cases [7]. Even though both ophthalmic and cerebrovascular manifestations of leukemia have been well established in the literature, their co-occurrence have not been well presented. Evidently, managing such cases requires multidisciplinary cooperation and poses a significant challenge for ophthalmologists. Herein, we aim to present a challenging case of concurrent bilateral subhyaloid hemorrhage and SAH in a patient with AML.

## Case presentation

A 36-year-old Persian male was first referred to our hospital because of increasing fatigue, pallor, scattered petechiae, and abnormal gum bleeding. The patient had been otherwise healthy and did not report any past medical conditions. Anemia and thrombocytopenia in initial lab results prompted the hematologist–oncologist to obtain peripheral blood smear (PBS). Upon spotting blasts in the PBS, the hematologist–oncologist requested flow cytometry and bone marrow biopsy analysis, which subsequently confirmed the diagnosis of acute monocytic leukemia (AML-M5). The patient was then started on four sessions of induction chemotherapy, using cytosine arabinoside in combination with daunorubicin in the first session and cytosine arabinoside in combination with idarubicin for the following sessions. The last induction chemotherapy session took place 3 months after the initial diagnosis, after which it was concluded that the patient’s disease had entered remission phase.

Ten days following the last chemotherapy session, the patient presented to the emergency room with complaints of fever, headache, and blurred vision. The patient reported that he experienced fever and chills in the previous 2 days and it was poorly controlled by using acetaminophen. His headache in the frontal region was persistent, moderate in intensity, and started with a sharp, stabbing characteristic but became dull over time. His vital signs, apart from a 39.5 °C fever, were stable. The patient appeared pale and scattered petechiae on the upper extremities were observed. No neurological deficit was detected. The patient was admitted to the internal medicine service. Initial lab results revealed pancytopenia including severe neutropenia [total white blood cell (WBC) count 300 × 10^9^/L], anemia [hemoglobin (Hg) 4.1 g/dL], and thrombocytopenia [platelets (Plt) 7000 10^9^/L]. C-reactive protein (CRP) and erythrocyte sedimentation rate (ESR) were significantly elevated (176 mg/L and 130 mm/hour, respectively). Coagulation parameters prothrombin time (PT), partial thromboplastin time (PTT), and international normalized ratio (INR) were normal (12 seconds, 25 seconds, and 1 respectively).

**Fig. 1:**
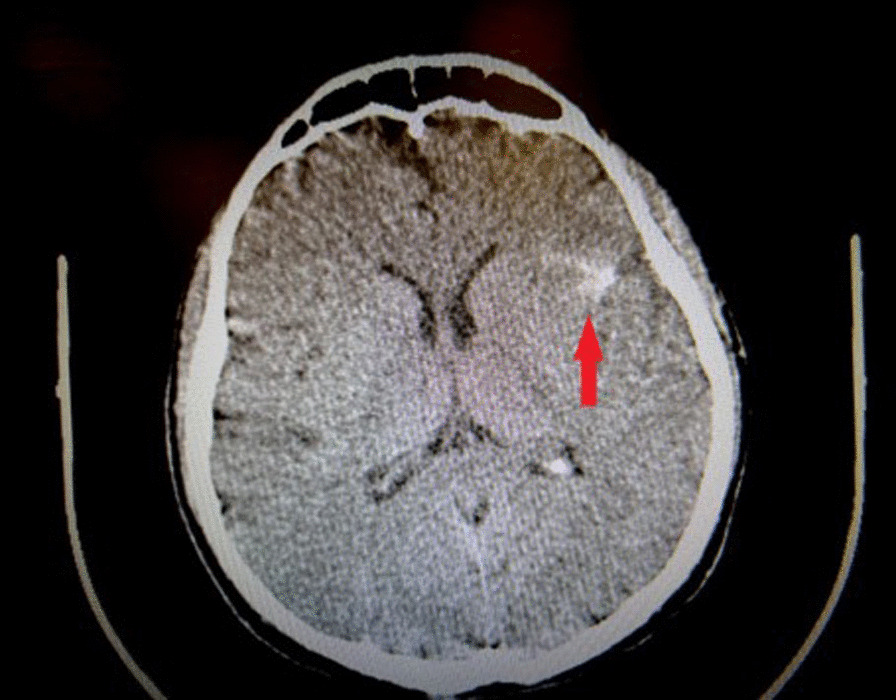
Axial cut of brain CT scan. Red arrow pointing to the SAH in left frontal and parietal lobes

Spinal brain computed tomography (CT) scan was carried out, which indicated subarachnoid hemorrhage (SAH) in the left frontal and parietal lobes of the brain as shown in Fig. 1. Midline shift and intraventricular hemorrhage were not detected. Neurosurgery was consulted and they concluded that no intervention would be necessary because of the self-limited nature of patient’s SAH and absence of neurological deficit.

**Fig. 2:**
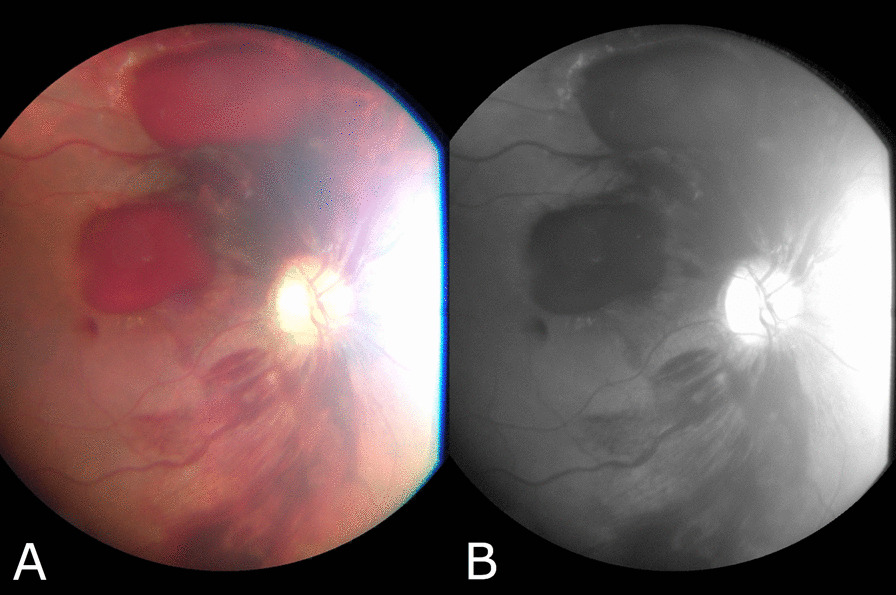
Fundus image of the right eye showing subhyaloid and intraretinal hemorrhage. **A** Color image **B** Red-free image

**Fig. 3:**
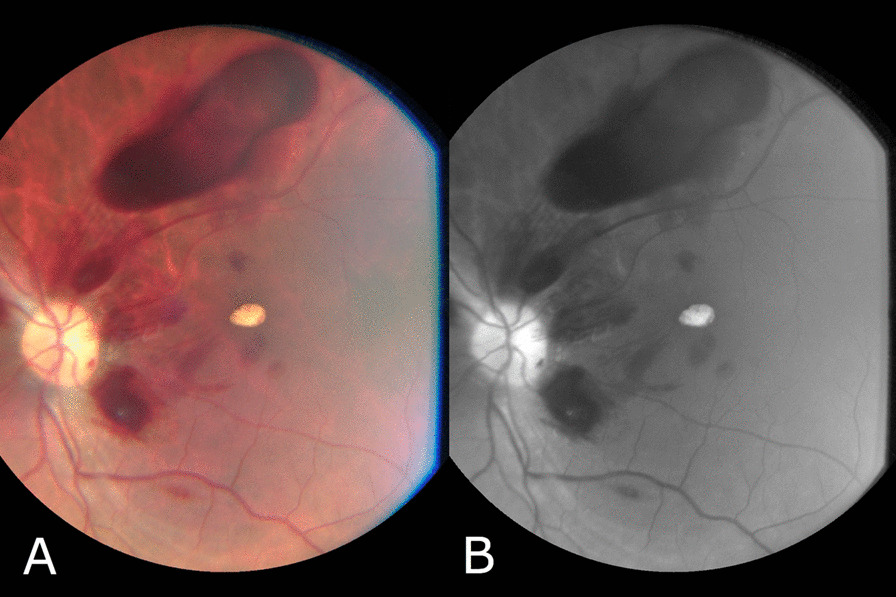
Fundus image of the left eye showing peripapillary subhyaloid and intraretinal hemorrhages. **A** Color image **B** Red-free image

Due to the patient’s blurred vision, ophthalmology consult was requested. We performed a thorough ophthalmic examination. The patient’s visual acuity consisted of light perception in the right eye and 3-m finger count in the left eye. Pupils of both eyes were midsize and briskly reactive, and no relative afferent pupillary defect was detected. Intraocular pressure (IOP) of both eyes were within normal limits. Conjunctiva and sclera were white and quiet. Cornea was clear. Anterior chamber was deep and quiet, the lenses were clear, and no vitreous hemorrhage was detected. Overall, anterior segment examination was unremarkable. Dilated fundus examination was performed. As shown in Fig. 2, in the right eye subhyaloid hemorrhage along with peripapillary intraretinal and flame-shaped hemorrhages were observed. Subhyaloid and intraretinal hemorrhages, along with a yellowish dense exudate in the fovea, is apparent in the fundus image of the left eye, as shown in Fig. 3. These findings, along with the patient’s underlying pancytopenia as a result of leukemia, confirmed leukemic retinopathy.

Given the patient’s complicated condition, treating the underlying hematologic disturbance that caused SAH and retinal hemorrhages was our main treatment approach. The patient’s thrombocytopenia and anemia were improved by multiple transfusions of platelets and packed cells. He was also started on antibacterial, antifungal, and antiviral medications for neutropenic fever. The patient’s vision did not improve during the course of admission; however, he did not develop any neurologic deficit and his retinal hemorrhage appeared to be limited. Therefore, we decided to continue conservative treatment and observe the patient through regular follow-ups. As a result, vitrectomy or hyaloidotomy was not performed. The patient’s SAH was reduced, no neurological deficit was observed, and further neurosurgical intervention was deemed not necessary. He was discharged after a successful treatment of neutropenic fever and correction of thrombocytopenia and anemia.

**Fig. 4:**
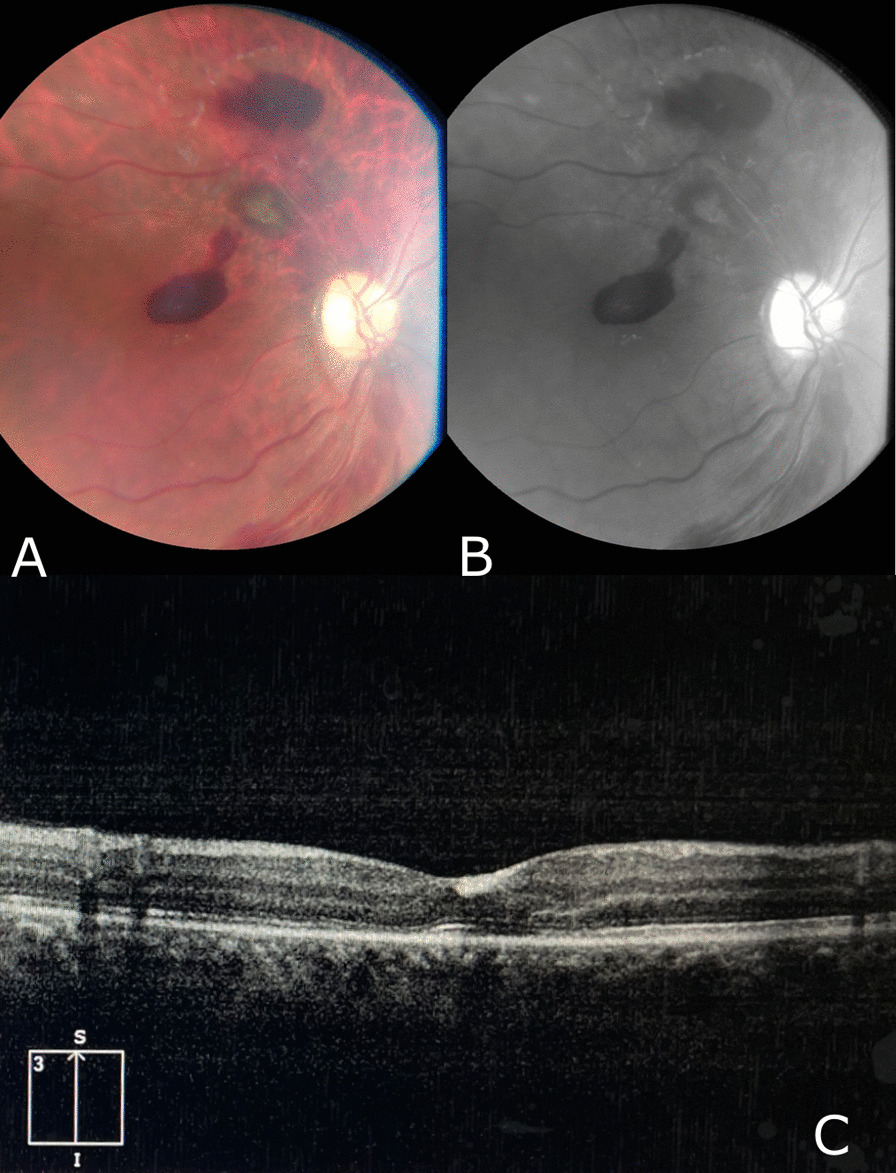
Fundus image of the right eye after 3 months of follow-up showing significant shrinkage and improvement of the subhyaloid hemorrhage. **A** Color Image **B** Red-free image **C** OCT image showing macular edema

**Fig. 5:**
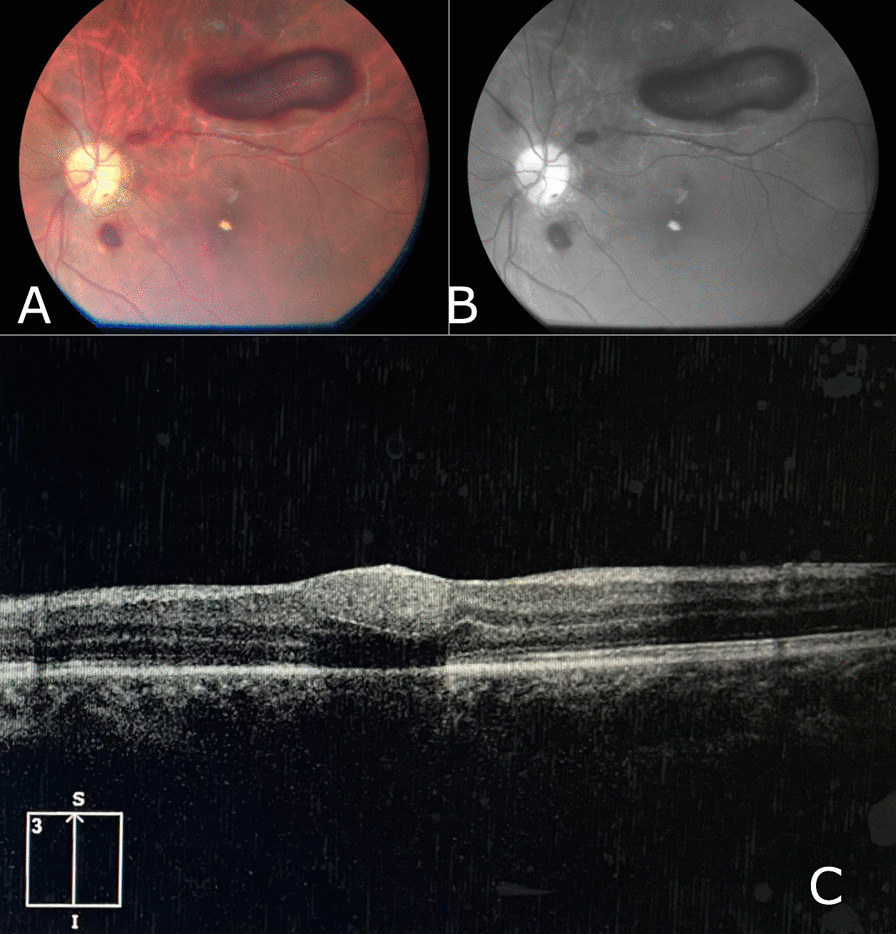
Fundus image of the left eye after 3 months of follow-up showing significant shrinkage and improvement of the subhyaloid hemorrhage and macular exudate. **A** Color image **B** Red-free image **C** OCT image showing exudate on the macula

We followed up with the patient for a duration of 3 months, during which he has shown significant improvement in his vision and retinal hemorrhage; however, complete resolution has not yet been achieved. The last fundus examination along with OCT are shown in Figs. 4 and 5. Shrinkage of retinal hemorrhages are observed in both eyes. Bilateral macular edema is also detected in OCT pictures.

## Discussion

Ophthalmic and cerebrovascular manifestations of leukemia have been separately and extensively studied in the literature. However, there has not been sufficient reporting of their co-occurrence. Lorenzi *et al*. reported a case of Terson syndrome in a patient with chronic leukemia [8]. Terson syndrome is defined as development of intraocular hemorrhage as a result of intracranial hemorrhage. Similarly, both intraocular and intracranial hemorrhages are present in our case. However, there is no evidence to indicate that subhyaloid hemorrhage was caused by SAH in this patient. Pancytopenia seems to be the underlying mechanism of both hemorrhages and one is not caused by the other.

Deciding on an appropriate treatment approach for ophthalmic manifestations of patients with leukemia is challenging and requires careful consideration, risk assessment, and interdisciplinary cooperation. In this case, given the patient’s poor state of health and underlying hematologic disturbances, such as severe thrombocytopenia, anemia, and neutropenia, any interventional procedure including laser or surgical procedure would most likely result in failure. As a result, a conservative follow-up approach was taken for the patient’s retinal hemorrhage. There has been extensive evidence in the literature that suggests that all patients with leukemia should be examined and monitored for ophthalmic manifestations. Mirshahi *et al*. indicated that 32% of patients newly diagnosed with leukemia had ophthalmic manifestations, out of which 90% were asymptomatic; thus pointing at the importance of regular eye examinations [9].

## Conclusions

This case properly highlights the extent of complications that some patients with leukemia may present with. Management approach of ophthalmic manifestations of leukemia involves interdisciplinary cooperation, and more importantly, should be individualized on the basis of the patients’ underlying medical condition.

## Data Availability

Not applicable.
